# The use of mucoadhesive oral patches containing epigallocatechin-3-gallate to treat periodontitis: an in vivo study

**DOI:** 10.1016/j.jtumed.2022.06.006

**Published:** 2022-07-07

**Authors:** Dur Muhammad Lashari, Mohammed Aljunaid, Yasmeen Lashari, Huda Rashad Qaid, Rini Devijanti Ridwan, Indeswati Diyatri, Nejva Kaid, Baleegh Abdulraoof Alkadasi

**Affiliations:** a Doctoral Program of Dental Medicine, Faculty of Dental Medicine, Universitas Airlangga, Surabaya, Indonesia; b Faculty of Oral Biology Dental Department Bolan Medical College Quetta, Pakistan; c Department of Oral and Dental Medicine, Faculty of Medicine, Taiz University, Taiz, Yemen; d Doctoral Program, Faculty of Medicine, Universitas Airlangga, Surabaya, Indonesia; e Bolan Medical College & Hospital Quetta, Department of Microbiology (Pathology), Quetta, Pakistan; f Faculty of Oral and Dental Medicine, AL-saeed University, Taiz, Yemen; g Department of Oral Biology, Faculty of Dental Medicine, Universitas Airlangga, Surabaya, Indonesia; h Master Program of Drug and Cosmetic Manufacturing Technology, Department of Pharmaceutical Technology, Faculty of Pharmacy, Yeditepe University, Istanbul, Turkey; i Head of Oral Medicine and Periodontology Department, Faculty of Dentistry, Ibb University, Ibb, Yemen

**Keywords:** فقدان العظام السنخية, إيبيغالوكاتشين, رقعة فموية, التهاب اللثة, بكتيرياالبورفيروموناس, يبيغالوكاتشين 3-جالاتي, Bone loss, Epigallocatechin-3-gallate, Oral patch, Periodontitis, *Porphyromonas gingivalis*

## Abstract

**Objectives:**

The application of topical drugs such as mucoadhesive oral patches (MOPs) do not irritate the mucosa and are able to increase the permeability of drugs to oral tissue. Epigallocatechin-3-gallate (EGCG) is an active ingredient that exhibits significant antibacterial and anti-inflammatory effects. The purpose of this study was to analyze the therapeutic potential of a mucoadhesive oral patch containing EGCG (MOP-EGCG) in a model of periodontitis and investigate its effects on the expression of osteoprotegerin (OPG), receptor activator of nuclear factor-kappa Β ligand (RANKL) and receptor activator of nuclear factor-κB (RANK).

**Methods:**

A model of periodontitis was induced in *Rattus novergicus* used *Porphyromonas gingivalis* by applying 0.03 ml of bacteria locally with 1 × 10^10^ colony-forming units (CFU) seven times at 2-day intervals in the central lower incisors. Periodontitis was then treated with MOP (control), a mucoadhesive oral patch containing doxycycline (MOP-doxy) or MOP-EGCG for 1 h/day for 21 days. On days 3, 5, 7, 14 and 21 after treatment, the central lower incisor was biopsied and analyzed by immunohistochemistry for RANK/RANKL and OPG expression in the gingiva tissue.

**Results:**

MOP-EGCG extract significantly reduced the expression of RANKL and increased the expression of OPG and RANK (p < 0.05) when compared to the MOP-doxy and MOP groups.

**Conclusion:**

MOP-EGCG extract reduced the expression of RANKL and increased the expression of OPG and RANK, thus suggesting that MOP-EGCG can inhibit the loss of alveolar bone in periodontitis.

## الملخص

**أهداف البحث:**

تطبيق الدواء الموضعي مثل الرقعة الفموية المخاطية لا يهيج الغشاء المخاطي وقادر على زيادة نفاذية الأدوية إلى أنسجة الفم. المركب المعروف باسم "إي جي سي جي" عبارة عن مكونات نشطة لها تأثيرات عالية مضادة للجراثيم ومضادة للالتهابات. كان الغرض من هذه الدراسة هو تحليل الإمكانات العلاجية للرقعة المخاطية الفموية التي تحتوي على مركب "إي جي سي جي" في نموذج التهاب اللثة وتأثيره على أوستيوبروتيجيرين (أو بي جي)، ومنشط مستقبلات لجين العامل النووي كابا-ب (رانكل)، ومنشط المستقبلات للعامل النووي كابا-ب (رانك).

**طرق البحث:**

استخدم نموذج التهاب اللثة المحرض في الجرذان النرويجية باستخدام 0.03 مل من بكتيريا "وحيدات الخلية البورفيرينية اللثوية" بفواصل زمنية لمدة يومين في القواطع المركزية السفلية. تم علاج التهاب اللثة لدى الفئران بـالرقعة الفموية المخاطية (مجموعة التحكم)، أو الرقعة الفموية المخاطية المحتوية على الدوكسيسيكلين، أو الرقعة الفموية المخاطية المحتوية على مركب "إي جي سي جي" لمدة 1 ساعة / يوم لمدة 5 أيام. في الأيام 3 و 5 و 7 و 14 و 21 بعد العلاج، تم أخذ عينة من القاطع المركزي السفلي وتحليله باستخدام الكيمياء المناعية لكل من "أو بي جي" و"رانكل" و"رانك"، في أنسجة اللثة.

**النتائج:**

بعد العلاج، زادت تعبيرات "رانك" و "أو بي جي" في جميع المجموعات مع أعلى زيادة في المجموعة المعالجة بمستخلص "إي جي سي جي" مقارنة بالمجموعتين الأخريين. كما أظهرت التحليلات انخفاضا في تعبيرات رانكل في جميع المجموعات بعد العلاج في الأيام 3 و 5 و 7 و 14 و 21. وكان للعلاج باستخدام مستخلص "إي جي سي جي" أقل تعبيرات رانكل في جميع الأيام مقارنة بالمجموعات الأخرى. أظهرت نتائج الاختبار اللاحق عدم وجود فرق ذي دلالة إحصائية بين مجموعة التحكم ومجموعة الدوكسيسيكلين في التعبير عن "أو بي جي" أو"رانكل" أو"رانك".

**الاستنتاجات:**

يمكن أن يقلل مستخلص"إي جي سي جي" من تعبير رانكل ويزيد "أو بي جي" و"رانك". من المفترض أن مستخلص"إي جي سي جي" قادر على منع فقدان العظام السنخية في التهاب اللثة.

## Introduction

Periodontitis refers to inflammation of the periodontal tissues and is characterized by alveolar bone resorption.^1^ Periodontitis is one of the most common infectious diseases and is derived from largely uncharacterized communities of oral bacteria that grow as biofilms on teeth and gingival surfaces in periodontal pockets. The current Global Burden of Disease Study reports that periodontitis is the sixth most prevalent global disease with an overall prevalence of up to 20% of the world's population.^2^ The inflammatory response of immune cells is activated by bacterial invasion. In later stages of disease, this causes the loss of both soft and hard supporting structures of the teeth. Thus far, only a few types of bacteria have been categorized as infectious agents of periodontitis.^3^ The successful treatment of periodontitis inhibits tissue destruction, stimulates osteointegration and inhibits osteoclastogenesis. Osteointegration and osteoclastogenesis include the activation and interaction of receptor activator of nuclear factor-κB (RANK), receptor activator of nuclear factor-kappa Β ligand (RANKL), and osteoprotegerin (OPG). Consequently, these proteins are critical for hemostatic control.^4^

As compared to other drug administration options, the mucoadhesive drug delivery system represents a much safer option. This system has many benefits compared to other administrative techniques. This method provides us with a controlled method for the release of drugs at a specific target site with extended retention times at the target site. One important benefit of this type of system is that it avoids the first phase of metabolism.^5^ The process by which polymers adhere to biological substances with mucus or the epithelium surface is called mucoadhesion. A substrate containing a bio-adhesive polymer can support the transfer of drugs to target sites and control the release of drug dosage over an extended period. Studies of mucoadhesive polymers have indicated that this is a good method for mucoadhesion and identified some factors that can affect the mucoadhesive properties of a polymer. Both natural and synthetic polymers are used in the preparation of mucoadhesive oral patches.^6^

A mucoadhesive drug delivery system is a safer delivery system when compared to other drug administration routes and has several advantages over conventional administration methods. Hence, in this study, we analyzed the therapeutic potential of a mucoadhesive oral patch (MOP) containing EGCG (MOP-EGCG) in a periodontitis model induced by *Porphyromonas gingivalis* and investigated its effect on the expression of RANK/RANKL and OPG.

## Materials and Methods

### Materials

Epigallocatechin-3-gallate (EGCG) concentrate from green tea extract (*Camellia sinensis*) was obtained from Xi’An Rongsheng Biotechnology Co., Ltd. (Shaanxi, China). Polyethylene glycol (PEG) was obtained from Schuchardt OHG (Germany). PEG 4000 was obtained from SigmaAldrich (St. Louis, MO). Sodium carboxymethyl cellulose (CMC-Na) and propylene glycol (PEG) was obtained from Teknis (Indonesia) and doxycycline was obtained from Kimia Pharma.

### Preparation of mucoadhesive oral patches

Mucoadhesive oral patch (MOP) preparation was performed using the solvent casting technique. Patches were prepared using CMC-Na and PG was used as a plasticizer to improve the performance and release characteristics of the patch.

CMC-Na (0.6 g) was dissolved in 30 ml of warm distilled water; then PG (2.5 g) was added under continuous stirring to obtain a suitable viscosity dispersion. The mixture has been poured into Petri dishes, stored at 4 °C for 48 h to remove entrapped air bubbles, and finally oven-dried at 40 °C. All patches were uniform, homogeneous, and free from bubbles; the thickness of the patch was 0.3 mm.

### Preparation of mucoadhesive oral patches containing EGCG and mucoadhesive oral patches containing doxycycline

The process used to create MOP-EGCG and MOPs containing doxycycline (MOP-doxy) were similar to those used for the preparation of blank patches. We dissolved CMC-Na (0.6 g) in 30 ml of warm distilled water with manual stirring. Then, EGCG (100 mg) and doxycycline (100 mg) were added to the CMC-Na solution separately and stirring was continued until the mixture became homogeneous. Then, PEG (1 g) was added with continuous stirring until suitable viscosity dispersion was obtained. The mixtures were then poured into Petri dishes and oven-dried at 40 °C. The final thickness of the patch was 0.3 mm and contained 15 mg EGCG and 3 mg doxycycline.

### Experimental model

The study protocol was approved by The Faculty of Veterinary Medicine, Airlangga University Laboratory. This was a laboratory experimental study with a post-test-only control group design. In total, we used 45 healthy male Wistar rats *(Rattus norvegicus),* aged between 5 and 6 months with a body weight of 250–300 g; all rats were characterized by energetic movement and were acclimatized for 1 week prior to experimentation. The experimental animals were received by the Faculty of Veterinary Medicine, Airlangga University Animal Laboratory. According to Lemeshow,^7^ a sample of 45 models was required, with each group containing three animals for a total of 15 groups.

### Periodontitis model

*P. gingivalis* (ATCC 33 277) was produced in media containing tryptic soy broth (TSB) and then raised in anaerobic conditions for 18–24 h. Bacterial colonies were taken and transferred in 3 ml of BHI broth media then incubated at 37 °C for 18 h. The bacterial suspension was synchronized with a 0.5 McFarland standard (10^10^ colony-forming units (CFU)/ml) and taken with a micropipette and dropped onto the surface of Mueller-Hinton agar media and incubated for 24 h at 37 °C in a 5% CO_2_ anaerobic atmosphere.^8^^,^^9^

The periodontitis model was achieved by injecting *P. gingivalis* (0.03 ml containing 1 × 10^10^ CFU) into the mandibular gingival incisive sulcus of the anterior teeth of animals seven times at 2-day intervals using a disposable syringe (0.5 cc).

### Periodontitis treatment application

Once periodontitis had developed, each group of animals received treatment after been anesthetized with 10% ketamine (0.1 ml/100 g body weight) delivered by intramuscular injection. Each group received MOP-EGCG, MOP-doxy or a blank patch for 1 h/day for 5 days. The patch was cut and sized into small pieces to fit the incisive gingival mucosa of the rat's jaw. Animals were killed on days 3, 5, 7, 14 and 21 after the commencement of treatment. Ketamine (0.4 ml/100 g body weight) by intramuscular injection was used as an anesthesia agent. The lower anterior incisive region of the mandible was biopsied.

### RANK/RANKL expression

RANK is a protein expressed in the osteoclasts of alveolar bone tissue. We used an anti-RANK (Biogear Indonesia) monoclonal antibody to semi-qualitatively investigate the expression of RANK/RANKL in experimental animals by immunohistochemical (IHC) methods with a light microscope (400× magnification); positive staining was seen as a brown color.

### OPG expression

OPG is a protein expressed in the osteoblasts of alveolar bone tissue. We used an anti-OPG (Biogear Indonesia) monoclonal antibody to semi-qualitatively investigate the expression of OPG by IHC methods with a light microscope (400× magnification); positive staining was seen as a brown color.

### Statistical analysis

Data analyses were conducted with SPSS (version 25). Data is shown in the form of mean ± standard deviation (SD). Normality tests were conducted with the Kolmogorov Smirnov test and differences between groups were tested by one-way analysis of variance and Tukey's post hoc test. A statistically significant difference was defined as p < 0.05.

## Results

### RANK expression

**Figure 1:**
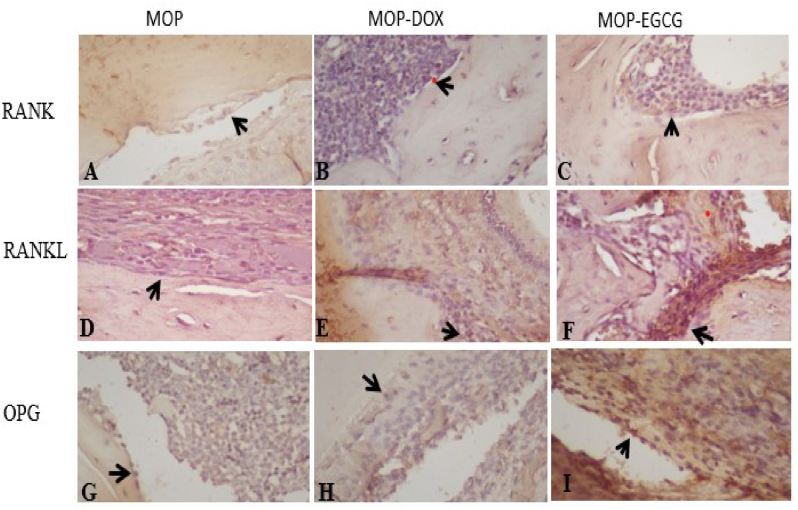
Immunohistochemical staining of RANK/RANKL and OPG expression in periodontitis.

**Figure 2:**
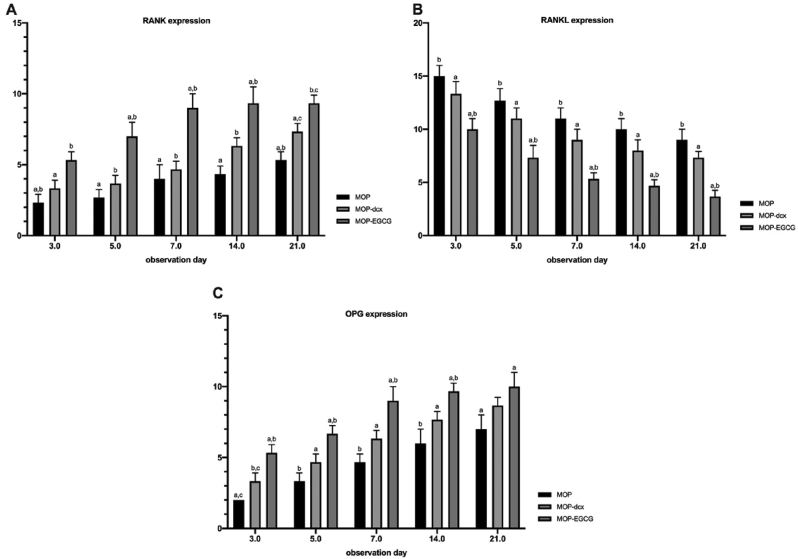
Expression levels of RANK, RANKL and OPG. The same letter on each a day of observation indicates significant differences, as determined by the Tukey-HSD test (p < 0.05).

IHC analyses revealed increased RANK expression in all groups post-treatment on days 3, 5, 7, 14 and 21; the MOP group had the lowest levels of RANK expression (Figure 2a). When compared with other groups, treatment with MOP-EGCG extract resulted in the highest expression levels of RANK on days 3, 5, 7, 14 and 21 (Figure 1, Figure 2). Tukey's post hoc test results showed that there were significant differences in RANK expression between the MOP and the MOP-EGCG group after 3, 5, 7, 14 and 21 days (p < 0.002, p < 0.001, p < 0.001, p < 0.001, and p < 0.001, respectively) and between the MOP and the MOP-doxy group after day 21 (p = 0.013). However, there was no significant difference in RANK expression between the MOP and MOP-doxy groups after 3, 5, 7 and 14 days (p = 0.165, p = 0.300, p = 0.645, and p = 0.054 respectively).

### RANKL expression

IHC analyses revealed a reduction in the expression levels of RANKL in all groups post-treatment on days 3, 5, 7, 14 and 21 (Figure 1, Figure 2b). Treatment with MOP-EGCG resulted in the lowest RANKL expressions on days 3, 5, 7, 14 and 21 when compared to the other groups (Figure 1, Figure 2). Tukey's post hoc test results showed that there were significant differences in the expression of RANKL between the MOP and MOP-EGCG groups after 3, 5, 7, 14 and 21 days (p < 0.003, p < 0.003, p < 0.001, p < 0.001, and p < 0.001, respectively). However, there were no significant differences in expression between the MOP and the MOP-doxy groups after 3, 5, 7, 14 and 21 days (p = 0.209, p = 0.234, p = 0.072, p = 0.072, and p = 0.075, respectively).

### OPG expression

IHC analyses revealed an increased in the expression of OPG in all groups post-treatment on days 3, 5, 7, 14 and 21. The MOP group had the lowest levels of OPG expression (Figure 1, Figure 2c). Treatment with MOP-EGCG led to the highest expression levels of OPG on days 3, 5, 7, 14 and 21 when compared to the other groups (Figure 1, Figure 2). Tukey's Post hoc testing showed that there were significant differences in the expression of OPG between the MOP and MOP-EGCG groups after 3, 5, 7, 14 and 21 days (p < 0.001, p < 0.001, p < 0.001, p < 0.002, and p < 0.014, respectively). However, there were no significant differences in expression between the MOP and the MOP-doxy groups after 3, 5, 7, 14 and 21 days (p = 0.075, p = 0.067, p = 0.075, p = 0.075, and p = 0.129, respectively).

## Discussion

This study aimed to determine the levels of RANK/RANKL and OPG in healed tissue models of periodontitis. The results revealed a significant increase in RANK and OPG expression, as well as a significant decrease in the expression of RANKL in a rat model of periodontitis. Our findings indicate the treatment of periodontitis led to a decrease in number of RANKL receptors but a significant increase in both OPG and RANK. Moreover, the number of RANK receptors increased; however, due to the reduction in the number of RANKL receptors, it was not possible for RANK to fully bind and activate the process of osteogenesis; instead, RANKL bound with OPG in order to bind with RANK. Overall, this process would lead to a reduction in the process of bone resorption.

An imbalance in the RANKL/OPG signaling pathway during osseointegration, defined by high expression levels of RANKL/OPG, might cause a disruption in tissue wound healing, thus increasing the risk of periodontitis. The osteoclastogenic profile of periodontitis has been documented in many previous studies.^10^^,^^11^ Prostaglandin E2 (PGE2), interleukin-1-beta (IL-1β), interleukin-6 (IL-6), and tumor necrosis factor-alpha (TNF-α), in combination with some other mediators of bone metabolism (transforming growth factor-beta [TGF-β], parathyroid hormone, 1,25-dihydroxyvitamin D_3_, glucocorticoids, and estrogen) have been shown to cause effects on osteoclastogenesis by regulating the osteoblastic/stromal cell production of OPG and RANKL and were detected immediately at infected periodontal sites.^12^ Bone loss has also been reported in periodontitis; a previous study showed that changes in the levels of RANKL play a vital role in bone loss.^13^^,^^14^ A rise in the expression levels of RANKL, along with a reduction of OPG expression, have been observed in gingival crevicular fluid.^15^

Herbal medicine is an alternative option for treatment and is not associated with side effects; consequently, herbal options are becoming increasingly popular. It has been verified that green tea extract exerts antibacterial activity against *P. gingivalis* bacteria, the most common pathogenic bacteria that cause periodontitis. Green tea is one of the most commonly used beverages worldwide and is obtained from the dried leaves of the plant *Camellia sinensis*.^16^ The polyphenol epigallocatechin-3-gallate is the most important of the catechin components of green tea.^18^ Green tea has been demonstrated to have scientifically proven benefits and many functional properties; at present, the consumption of green tea is widely recommended.^17^ Previous studies have investigated how the topical application of green tea extract gel might reduce infection in the periodontium, thus suggesting that the formulation could be used as an adjuvant treatment for regeneration after periodontitis.^19^

Analysis of RANKL showed that there were significant differences between the negative control group and the positive control group; the treatment group and the negative control group; and the positive control group and the treatment group. Analysis of OPG revealed that there were significant differences between the control negative group and the positive control group and the treatment group; and between the control negative group, positive control group and the treatment group.

In a previous in silico study, we use EGCG compounds as a ligand for formulas obtained from a Pub Chem database. Targeted compounds of three-dimensional (3D) structures underwent energy minimization at PyRx to stabilize the molecular structure and acquire a bank of data.^20^ A number of proteins were identified in this previous study, including sclerostin, TRAP, Rank-rank, osteocalcin, NFATC1, osterix, and RUNX2; these proteins were identified by the RCSB PDB database. The 3D structures were then downloaded in the pdb protein format; then, we sterilized the water molecules and native ligand through the PyMol software. EGCG binding occurred with NFACT, sclerostin TRAP, RANK-RANKL, thus demonstrating inhibitory effects.^21^^,^^22^

There were some limitations to this study that need to be considered. For example, because we needed a long contact time of 1 h between the mucoadhesive gingival patch and the gingiva, we needed to apply an anesthetic with a long duration of action. Furthermore, because the patch slides easily from the gingival incisive sulcus, it needs to be held or tied with a ligature. However, despite of these limitations, the results of this study confirm that the use of EGCG in MOP can play a role in the treatment of periodontitis, in which RANK, RANKL and OPG expression levels can be used as the main indicators of periodontitis. To strengthen our findings, further research is required. For example, we need to identify the precise effects of MOP-EGCG on other markers of periodontitis such as TNF-α and IL-10. We also need to investigate other subspecies of MOP to evaluate the antibacterial activity against periodontopathic bacteria and apply other products such as gels, mouthwashes and toothpaste that be formulated with EGCG.

## Conclusion

Mucoadhesive oral patches loaded with epigallocatechin-3-gallate extract prevented alveolar bone damage in a model of periodontitis by inhibiting the expression of RANKL and increasing the expression of OPG and RANK.

## Source of funding

This study was supported by the 10.13039/501100017022Ministry of Higher Education, the Republic of Indonesia in Scheme Penelitian Disertasi Doktor (PDD) 2021 (Grant No. 275/UN3/2021).

## Conflict of interest

None of the authors have any conflicts of interest to declare.

## Ethical approval

This study was performed in strict accordance with the Guide for the Care and Use of Laboratory Animals, National Health Research and Development Ethics Standard and Guidelines Council (2017), Minister of Health, Republic of Indonesia. This research study obtained ethical approval (Reference 466/HRECC.FODM/X/2020, Approval Date: 9 October 2020) from the Research Ethics Commission of the Faculty of Dentistry, Airlangga University, Surabaya.

## Author contributions

DML, MA, and HRQ carried out the research and collected the data. RDR designed and supervised the study, visualized, and validated the data, acquired funding, and reviewed draft material. The data were organized, analyzed, and interpreted by ID and BAA along with YL and NQ who also reviewed the article. All authors have critically reviewed and approved the final draft and are responsible for the content and similarity index of the manuscript..
